# p16^INK4a^ and pRb expression in laryngeal squamous cell carcinoma with and without infection by EBV or different genotypes of HPV: a retrospective study

**DOI:** 10.1186/s13027-023-00514-x

**Published:** 2023-07-11

**Authors:** Jose Manuel Vazquez-Guillen, Gerardo C. Palacios-Saucedo, Alondra Yamileth Alanis-Valdez, Andrea Huerta-Escobedo, Angel Zavala-Pompa, Lydia Guadalupe Rivera-Morales, Ana Carolina Martinez-Torres, Vianey Gonzalez-Villasana, Julio Cesar Serna-Hernandez, Silvia Judith Hernandez-Martinez, Edmundo Erbey Castelan-Maldonado, Martha Socorro Montalvo-Bañuelos, Cesar Alejandro Alonso-Tellez, Ethel Corinthia Sanchez-Fresno, Reyes S. Tamez-Guerra, Cristina Rodriguez-Padilla

**Affiliations:** 1 Laboratorio de Inmunologia y Virologia, Facultad de Ciencias Biologicas, Universidad Autonoma de Nuevo Leon, San Nicolas de los Garza, Nuevo Leon Mexico; 2 Division de Investigacion, Departamento de Otorrinolaringologia y Departamento de Anatomia Patologica, Unidad Medica de Alta Especialidad, Hospital de Especialidades No. 25, Instituto Mexicano del Seguro Social, Monterrey, Nuevo Leon Mexico; 3 Laboratorio Medicina Diagnostica S.A. de C.V., Monterrey, Nuevo Leon Mexico; 4 Departamento de Biologia Celular y Genetica, Facultad de Ciencias Biologicas, Universidad Autonoma de Nuevo Leon, San Nicolas de los Garza, Nuevo Leon Mexico; 5 Departamento de Foniatria, Hospital General de Zona No. 6, Instituto Mexicano del Seguro Social, San Nicolas de los Garza, Nuevo Leon Mexico

**Keywords:** p16 ^INK4a^, pRb, Human papillomavirus, Epstein–Barr virus, Laryngeal squamous cell carcinoma

## Abstract

**Background:**

Laryngeal squamous cell carcinoma (LSCC) represents one of the principal tumors of the head and neck. Human papillomavirus (HPV) and Epstein–Barr virus (EBV) are considered risk factors for the development and the clinical prognosis of LSCC. High levels of p16^INK4a^ are suggested as a surrogate marker of HPV or EBV infection in some head and neck tumors but in LSCC is still controversial. Furthermore, pRb expression may be considered an additional biomarker but it has not been clearly defined. This work aimed to compare the expression of pRb and p16^INK4a^ as possible biomarkers in tumor tissues with and without infection by EBV or different genotypes of HPV from patients with LSCC.

**Methods:**

Tumor samples from 103 patients with LSCC were previously investigated for the presence and genotypes of HPV using the INNO-LiPA line probe assay and for the infection of EBV by qPCR. p16 ^INK4a^ and pRb expression was assessed by immunohistochemistry.

**Results:**

Of the 103 tumor samples, expression of p16^INK4a^ was positive in 55 (53.4%) and of this, 32 (56.1%) were positive for HPV whereas 11 (39.3%) were EBV positive but both without a significantly difference (*p* > 0.05). pRb expression was positive in 78 (75.7%) and a higher frequency of this expression was observed in HPV negative samples (87.0%) (*p* = 0.021) and in high-risk HPV negative samples (85.2%) (*p* = 0.010). No difference was observed when comparing pRb expression and EBV infection status (*p* > 0.05).

**Conclusion:**

Our results support the suggestion that p16^INK4a^ is not a reliable surrogate marker for identifying HPV or EBV infection in LSCC. On the other hand, most of our samples had pRb expression, which was more frequent in tumors without HPV, suggesting that pRb could indicate HPV negativity. However, more studies with a larger number of cases are required, including controls without LSCC and evaluating other molecular markers to determine the real role of p16^INK4a^ and pRb in LSCC.

## Introduction

Laryngeal squamous cell carcinoma (LSCC) is one of the most common type of head and neck cancer and, although tobacco smoking and alcohol abuse are the principal risk factors, some viral infections are implicated [1, 2]. Human papillomavirus (HPV) is a group of oncoviruses that have been widely associated with the development and clinical prognosis of LSCC tumors [3]. According to their oncogenic potential, HPV are categorized into low-risk (LR-HPV) or high-risk (HR-HPV) genotypes [4]. LR-HPV are associated with warts or skin papillomas, whereas HR-HPV are directly associated with the development of invasive carcinomas [5, 6]. Prevalence studies have reported that HPV-16 and -18 are the most common HR-HPV genotypes found in LSCC although, other HR-HPV, such as 31, 33, 35, 39, 45, 51, 52, 56, 58, and 59 are also important [7, 8]. Epstein–Barr virus (EBV) is strongly associated with onset and progression of several types of epithelial cell tumors such as nasopharyngeal carcinoma or gastric adenocarcinoma [9, 10]. The role of EBV in LSCC is controversial since difficulties have been reported in detecting its presence in this kind of cancer or it has shown a low prevalence [11]. Both HPV and EBV produce oncoproteins that have the potential to induce processes of carcinogenesis and tumor progression [12]. E7 is an oncoprotein coded by HPV that promotes retinoblastoma protein (pRb) degradation by the ubiquitin–proteasome pathway [13]. Degradation of pRb consequently induces the overexpression of the tumor suppressor protein p16^INK4a^, which is essential in cell cycle progression [14]. Latent membrane protein 1 (LMP1) is one of the major EBV oncoproteins that inactivates transcription factors essential for the expression of p16^INK4a^ [15]. The alteration of p16 ^INK4a^ expression leads to hyperphosphorylation of pRb that promotes uncontrolled cellular proliferation [16]. High levels of p16^INK4a^ expression have been suggested as a surrogate marker of HPV and EBV infection in pharyngeal, nasopharyngeal, and oropharynx tumors, whereas in laryngeal tumors are still controversial; therefore, pRb expression may be considered as an additional biomarker, particularly in tumors with infection by HPV and EBV [12, 17–19]. This work is aimed to compare the expression of pRb and p16^INK4a^ as possible biomarkers in tumor tissues with and without infection by EBV or different genotypes of HPV from patients with LSCC attending a third-level care referral hospital.

## Materials and methods

### Samples

Formalin-fixed and paraffin-embedded (FFPE) tissue specimens from patients with LSCC were included in a retrospective study. Samples were taken from a previous study evaluating the HPV and EBV prevalence in patients with LSCC [20]. All patients had undergone surgical resection of the laryngeal tumor between 2012 and 2015 at the Unidad Medica de Alta Especialidad (UMAE) No. 25, a tertiary referral hospital of the Instituto Mexicano del Seguro Social (IMSS) located in northeastern Mexico. The information obtained from clinical records included age, sex, history of alcohol and tobacco consumption, and laryngeal subsite location of the tumor (subglottic, glottic, or supraglottic). Histological and pathological grading of tumors was made using the Broder’s classification [21]. The National Committee of Scientific Research of the IMSS granted ethical approval to carry out the study (R-2014-785-055). Individual informed consent was not obtained as specimens were retrospectively collected.

### HPV genotyping and EBV detection

Tumor sections from each FFPE sample were carefully punched off and conducted to a DNA isolation using the NucleoSpin DNA FFPE (Macherey–Nagel, Düren, Germany). The presence and genotype of HPV were evaluated using the INNO-LiPA HPV Genotyping Kit Extra II Amp (Innogenetics, Gent, Belgium), a line probe assay designed for the identification of 32 genotypes of the HPV, including thirteen HR-HPV (HPV-16, HPV-18, HPV-31, HPV-33, HPV-35, HPV-39, HPV-45, HPV-51, HPV-52, HPV-56, HPV-58, HPV-59, and HPV-68), six possible HR-HPV (HPV-26, HPV-53, HPV-66, HPV-70, HPV-73, and HPV-82), nine LR-HPV (HPV-6, HPV-11, HPV-40, HPV-42, HPV-43, HPV-44, HPV-54, HPV-61, and HPV-81), and other four not yet classified HPV genotypes (HPV-62, HPV-67, HPV-83, and HPV-89) [22]. To detect DNA of EBV, a 69 bp fragment flanking the 679–748 position of the viral LMP2 glycoprotein was amplified using the primers 5′-AGC TGT AAC TGT GGT TTC CAT GAC-3′ and 5′-GCC CCC TGG CGA AGA G-3′ with the probe 6FAM5′-CTG CTG CTA CTG GCT TTC GTC CTC TGG-3′TAMRA. All the reactions for EBV detection were performed after the HPV genotyping assays using the TaqMan Universal PCR MasterMix (Applied Biosystems, Foster City CA, USA) in the Light Cycler (Roche). Synthetic DNA fragment (gBlocks Gene Fragment, IDT) containing the 69 bp sequence of the amplicon of EBV was used as amplification control and to evaluate the efficiency and detection limits of the assay.

### p16^INK4a^ and pRb expression

Immunohistochemical (IHC) staining method for p16^INK4a^ and pRb was performed on 5-µm-thick FFPE tissue cross-sections using the mouse and rabbit specific HRP/DAB (ABC) detection IHC kit (Abcam, Cambridge, UK) with the monoclonal anti-CDKN2A/p16INK4a antibody (clone EPR1473, ab108349, dilution 1:100; Abcam, Cambridge, UK) and the monoclonal anti-Rb antibody (clone EPR17512, ab181616, dilution 1:250; Abcam, Cambridge, UK). Hematoxylin–eosin (HE) stains were also prepared for histological evaluation of tumors. Two independent pathologists who were blinded to the clinical information and to the HPV or EBV status of the samples evaluated all the IHC and HE stains. Evaluations were made based on staining intensity as other previous reports have done. Specimens were considered positive for p16^INK4a^ expression if they showed ≥ 75% of stained cells, whereas for pRb, ≥ 25% of stained cells were interpreted as a positive expression [23–26].

### Statistical analysis

We used the Mann–Whitney U test, Pearson's X^2^ test, and Fisher's exact test. A *p* value < 0.05 was considered statistically significant. All analyzes were performed using the IBM SPSS Statistics program (Version 20, SPSS. Inc., Chicago, USA).

## Results

**Fig. 1:**
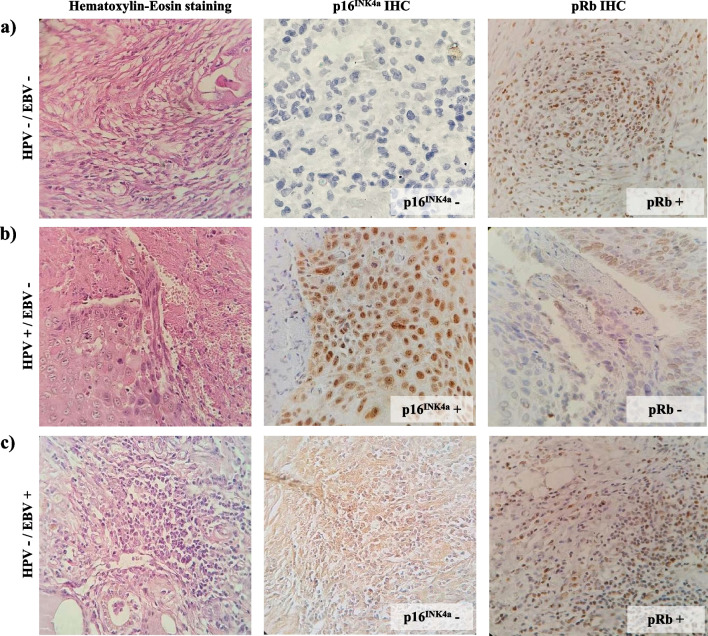
Representative examples of immunohistochemical (IHC) staining of tumors from patients with laryngeal squamous cell carcinoma. **a** Sample of patient No. 175 without infection of HPV or EBV; **b** sample of patient No. 046 that was positive for the 11 and 16 HR-HPV and negative for EBV; and **c** sample of patient No. 021 that was negative HPV and positive for EBV (× 40 microscopic magnification)

One hundred and three FFPE specimens from the previously published study with sufficient tumor tissue for IHC were eligible for the present work. The age range of the included patients was 40 to 89 years, with a median of 64, and most were 60 years or older (73.8%). Most samples were from men (96.1%), and tobacco smoking was common (98.0%). In contrast, alcohol intake frequency was low (6.8%). The glottis subsite was the most frequent location of tumors (49.5%). We had 57 (55.3%) tumor samples positive for HPV DNA, and 42 (40.8%) of these had at least one HR-HPV genotype. EBV DNA was present in 28 (27.2%) samples. Of these, 16 (15.5%) were in co-infection with HPV, 11 (10.7%) corresponded to co-infections with at least one HR-HPV, and 12 (11.7%) cases had only EBV infection (Table 1). Clinical characteristics of patients with LSCC tumor samples positive for at least one HR-HPV are summarized in Table 2. HPV-52 was the most frequently HR-HPV genotype, with 34 cases corresponding to 59.6% of all the HPV-positive samples. Immunohistochemistry were assessed considering a positive expression of p16^INK4a^ if ≥ 75% of tumor cells were stained and for pRb if ≥ 25% of stained cells were observed (Fig. 1).

**Table 1:** Correlation of p16^INK4a^ and pRb expression with clinicopathological characteristics of 103 patients with laryngeal squamous cell carcinoma

	Total (n = 103)	p16^INK4a^ positive^a^ (n = 55)	p16^INK4a^ negative^b^ (n = 48)	*p*	pRb positive^c^ (n = 78)	pRb negative^d^ (n = 25)	*p*
Age (years)	64 (40–89)	64 (43–87)	63.5 (40–89)	0.086	64 (40–89)	64.5 (46–81)	0.216
Age ≥ 60 years	76 (73.8%)	40 (52.6%)	36 (47.4%)	0.793	59 (77.6%)	17 (22.4%)	0.456
Sex				1.000			0.570
Male	99 (96.1%)	53 (53.5%)	46 (46.5%)		74 (74.7%)	25 (25.3%)	
Female	4 (3.9%)	2 (50.0%)	2 (50.0%)		4 (100%)	0 (0.0%)	
Surgical sample				0.009			0.025
Biopsy	57 (55.3%)	37 (64.9%)	20 (35.1%)		48 (84.2%)	9 (15.8%)	
Laryngectormy	46 (44.7%)	18 (39.1%)	28 (60.9%)		30 (65.2%)	16 (34.8%)	
Anatomic location				0.575			0.436
Glottic	51 (49.5%)	28 (54.9%)	23 (45.1%)		42 (81.4%)	9 (17.6%)	
Subglottic	4 (3.9%)	2 (50.0%)	2 (50.0%)		3 (75.0%)	1 (25.0%)	
Supraglottic	15 (14.6%)	10 (66.7%)	5 (33.3%)		11 (73.3%)	4 (26.7%)	
Transglottic	33 (32.0%)	15 (45.4%)	18 (54.6%)		22 (66.7%)	11 (33.3%)	
Hitopathological grade				0.010			0.421
Grade I	44 (42.7%)	22 (50.0%)	22 (50.0%)		35 (79.5%)	9 (20.5%)	
Grade II	38 (36.9%)	16 (42.1%)	22 (57.9%)		26 (68.4%)	12 (31.6%)	
Grade III	21 (20.4%)	17 (80.9%)	4 (19.1%)		17 (81.0%)	4 (19.0%)	
Smoking	101 (98.0%)	54 (53.5%)	47 (46.5%)	1.000	76 (75.2%)	25 (24.8%)	1.000
Alcoholism	7 (6.8%)	1 (14.3%)	6 (85.7%)	0.048	6 (85.7%)	1 (14.3%)	1.000
HPV infection				0.557			0.021
Postitive	57 (55.3%)	32 (56.1%)	25 (43.9%)		38 (66.7%)	19 (33.3%)	
Negative	46 (44.7%)	23 (50.0%)	23 (50.0%)		40 (87.0%)	6 (13.0%)	
HR-HPV infection				1.000			0.010
Positive	42 (40.8%)	22 (52.4%)	20 (47.6%)		26 (61.9%)	16 (38.1%)	
Negative	61 (59.2%)	33 (54.1%)	28 (45.9%)		52 (85.2%)	9 (14.8%)	
EBV infection				0.119			1.000
Positive	28 (27.2%)	11 (39.3%)	17 (60.7%)		21 (75.0%)	7 (25.0%)	
Negative	75 (72.8%)	44 (58.7%)	31 (41.3%)		57 (76.0%)	18 (24.0%)	
Coinfections							
HPV + EBV	16 (15.5%)	11 (68.8%)	5 (31.2%)	0.529	9 (56.3%)	7 (43.7%)	1.000
HR-HPV + EBV	11 (10.7%)	7 (63.6%)	4 (36.4%)	0.455	6 (54.5%)	5 (45.5%)	1.000

Values are shown in absolute frequencies (percent)

^a^≥ of 25% of staining cells

^b^< of 25% of staining cells

^c^≥ of 75% of staining cells

^d^< of 75% of staining cells

**Table 2:** Clinical data and status of p16^INK4a^ and pRb expression in 42 laryngeal squamous cell carcinoma patients positive for at least one high-risk human papillomavirus

Patient no.	Sex	Age	Anatomical location	Grade	Smoking	Alcoholism	HPV genotype	EBV	p16^INK4a^	pRb
007	M	≥ 60	Subglottic	II	Yes	Yes	11, 52	−	Negative	Positive
009	M	< 60	Transglottic	I	Yes	No	16	−	Negative	Negative
010	M	≥ 60	Glottic	I	Yes	No	52	−	Negative	Positive
013	M	≥ 60	Supraglottic	II	Yes	No	52	−	Positive	Positive
015	M	≥ 60	Glottic	III	Yes	No	16	−	Negative	Positive
029	M	≥ 60	Supraglottic	II	Yes	No	11, 16	−	Positive	Positive
045	M	≥ 60	Glottic	III	Yes	No	11, 52	−	Positive	Negative
046	M	≥ 60	Glottic	II	Yes	No	11, 16	−	Positive	Negative
047	M	≥ 60	Transglottic	III	Yes	No	6, 11, 52	−	Negative	Negative
049	M	≥ 60	Transglottic	II	Yes	No	11, 52	−	Negative	Negative
051	M	≥ 60	Supraglottic	I	Yes	No	6, 11, 52	−	Positive	Positive
052	M	≥ 60	Glottic	I	Yes	No	16, 52	−	Positive	Positive
053	M	≥ 60	Transglottic	I	Yes	No	11, 52	−	Negative	Negative
056	M	≥ 60	Glottic	II	Yes	No	11, 45	−	Negative	Negative
059	M	< 60	Transglottic	II	Yes	No	52	+	Positive	Negative
060	M	≥ 60	Glottic	II	Yes	No	11, 52	−	Positive	Positive
061	M	≥ 60	Glottic	I	Yes	Yes	11, 16	−	Negative	Positive
062	M	≥ 60	Transglottic	II	Yes	No	6, 11, 52	−	Negative	Positive
063	M	< 60	Subglottic	I	Yes	No	11, 31, 52, 54	+	Positive	Negative
067	M	< 60	Glottic	I	Yes	No	6, 11, 52	+	Positive	Positive
068	M	≥ 60	Transglottic	II	Yes	Yes	11, 16, 52	+	Negative	Positive
069	M	< 60	Transglottic	I	Yes	No	11, 52	−	Positive	Positive
070	M	≥ 60	Transglottic	II	No	No	11, 52	+	Positive	Positive
071	M	≥ 60	Transglottic	I	Yes	No	11, 31, 52, 54	−	Positive	Positive
072	M	≥ 60	Transglottic	I	Yes	No	11, 52	−	Negative	Negative
074	M	≥ 60	Supraglottic	II	Yes	No	11, 31, 52, 54	+	Negative	Negative
075	M	≥ 60	Glottic	III	Yes	No	11, 26, 31, 52, 54	+	Positive	Positive
079	M	≥ 60	Glottic	II	Yes	No	11, 31, 52, 54	−	Positive	Positive
080	M	< 60	Glottic	I	Yes	No	11, 52	−	Negative	Negative
084	M	< 60	Glottic	II	Yes	No	6, 11, 16, 52	−	Positive	Negative
085	M	≥ 60	Supraglottic	II	Yes	No	11, 52	−	Positive	Negative
088	M	≥ 60	Transglottic	II	Yes	No	6, 11, 52	+	Negative	Negative
089	M	≥ 60	Glottic	I	Yes	No	11, 52	−	Positive	Negative
090	M	≥ 60	Transglottic	I	Yes	No	11, 31, 52	+	Positive	Positive
091	M	≥ 60	Glottic	II	Yes	No	11, 52	−	Positive	Positive
092	M	≥ 60	Transglottic	I	Yes	No	11, 31, 52	−	Negative	Positive
097	M	< 60	Glottic	I	Yes	No	52	−	Negative	Positive
099	M	≥ 60	Supraglottic	III	Yes	No	52	−	Positive	Positive
124	F	≥ 60	Glottic	I	Yes	No	45	−	Positive	Positive
141	M	< 60	Glottic	II	Yes	No	52	−	Negative	Positive
148	M	≥ 60	Glottic	II	Yes	No	52	+	Negative	Positive
149	M	≥ 60	Glottic	II	No	No	45	+	Negative	Positive

Of the 103 tumor samples, p16^INK4a^ expression was positive in 55 (53.4%). According to the surgical source of the sample, biopsies (64.5%) had a higher frequency of expression of this protein than laryngectomies (*p* = 0.009 by the Pearson's chi-squared test). Samples cataloged as Grade III by Broder's classification showed a higher frequency of p16^INK4a^ expression (80.9%) compared with those of grade I (50.0%) and II (42.1%) (*p* < 0.05 by the Mann–Whitney U test). Only one of the seven alcohol consumers showed p16^INK4a^ expression (14.3%) (*p* = 0.048 by the Fisher's Exact Test). No significant difference was observed when comparing p16^INK4a^ expression with the presence of HPV or EBV (*p* > 0.05 by the Fisher's Exact Test). Regarding pRb expression, 78 (75.7%) samples were positive. Specimen’s obtained from biopsies (84.2%) showed a higher frequency of pRb expression than laryngectomy samples (*p* = 0.025 by the Pearson's chi-squared test). The frequency of pRb expression was higher in HPV negative samples (87.0%) (*p* = 0.021 by the Fisher's Exact Test). A higher frequency of pRb expression was also observed in the group of HR-HPV negative samples (85.2%) (*p* = 0.010 by the Fisher's Exact Test). No significant differences (*p* > 0.05) were observed in the expression of p16^INK4a^ or pRb by age and sex of patients, laryngeal tumor subsite, and tobacco consumption habits of the patients (Table 1).

## Discussion

To assess the expression of p16 ^INK4a^ and pRb in LSCC specimens with and without infection by EBV or different genotypes of HPV, 103 FFPE samples retrospectively collected from patients were evaluated by IHC analysis. The epidemiological characteristics of the studied sample are consistent with those previously described for this disease [11, 19, 27–33]. p16^INK4a^ status has been well characterized in HPV-positive head and neck tumors such oropharyngeal carcinoma and has been suggested as a surrogate marker for infection of HPV and progression, but this has not been definitively established for LSCC [34, 35]. In this study, 55 (53.4%) of 103 patients were positive for p16^INK4a^. Tong et al. found that 115 (54%) of 211 LSCC specimens were positive for p16^INK4a^ [36]. Elhadj et al. show that the expression positive of p16^INK4a^ was found in 36 (51.43%) of 70 LSCC cases [37]. Expression of p16^INK4a^ might be affected by genetic or epigenetic mechanisms [38]. Some studies suggest hypermethylation may represent an early event in carcinogenesis of different neoplasms, such as endometrial cancer [39]. In LSCC, expression level of p16^INK4a^ is significantly reduced and hypermethylation has been shown to be a common mechanism causing this downregulation [40]. We found that 80.9% of our specimens classified as grade III presented a higher p16^INK4a^ expression. In contrast to our results, Tong and et al. found that p16^INK4a^ expression was observed more commonly in well-differentiated samples [36]. In our study, only 7 (6.8%) patients were alcohol consumers, and one of them expressed p16^INK4a^. Although this low number of cases precludes reliable statistical comparison, most alcohol consumers were significantly negative for p16^INK4a^ expression (*p* = 0.048). Elhadj et al. found no association between alcohol consumption and p16^INK4a^ expression when evaluating 30 alcohol-consuming patients [37]. Otherwise, we found that 32 (56.1%) of the fifty-five positive samples for p16 ^INK4a^ were also positive for HPV infection. Stephen et al. reported the expression of p16^INK4a^ in 21 (26.0%) of 80 patients, furthermore, 12 (57.0%) of these were positive for HPV infection [34]. Hernandez et al. observed p16^INK4a^ expression in 8 (7.9%) of 101 cases, and only 2 (25.0%) of these were also HPV DNA positive [41]. Sanchez et al. found that 48 (39.0%) of 123 samples were positive for p16^INK4a^ expression, and 14 (29.2%) of these were positive for HPV infection [42]. Dogantemur et al. observed p16^INK4a^ expression in 18 (20.0%) of 90 cases and only 6 (33.3%) of these were also positive for DNA of HPV [43]. The fact that no association between the expression of p16^INK4a^ and HPV infection was observed could support the suggestion that this protein may not be reliable as a surrogate marker for HPV infection [44].

Modifications of the p16^INK4a^ and pRb expression have been delineated in several types of human cancers [45, 46]. We found that pRb expression was present in 78 (75.7%) of 103 specimens. Morshed et al. found that 90 (69%) of 130 patients with LSCC had expression of pRb [47]. Krecicki et al. reported that 51(88%) of 58 samples were positive for pRb expression [48]. Some studies suggest that high pRb expression may be due to different factors. Soares et al. suggested that it can be due to an increase in the proportions of proliferating cells, which is supported by the fact that the hyperphosphorylated pRb inactive form increases during the G2/M phases [49]. IHC evaluation using antibodies against phosphorylated and non-phosphorylated pRb forms could solve this. As pRb is a negative regulator of p16^INK4a^, its inactivation results in overexpression of p16^INK4a^. Thus, a positive test for HPV combined with p16^INK4a^ expression has been described as evidence of biologically relevant infection [50, 51]. The frequency of pRb expression was significantly higher in the HPV-negative specimens; we found 40 (87.0%) with positive pRb expression in 46 HPV-negative tumors. However, the clinical and prognostic significance remains poorly described in LSCC. Soares et al. found a significant difference between pRb expression and HPV-positive (9/11, 81.8%) and HPV-negative (15/22, 68.2%) oral squamous cell carcinoma (OSCC) samples [49]. Shaikh et al. demonstrated that pRb expression was predominant in 51 (77.3%) of 66 HPV-negative head and neck cancers cases, but they did not find significant differences [51]. We also observed a significantly higher frequency of pRb expression in HR-HPV negative specimens. The pRb expression was present in 51 (85.2%) of 61 HR-HPV negative samples. By contrast, Nemes et al. reported no statistical difference in 37 (84%) of 44 HR-HPV negative OSCC samples with pRb expression [52]. Various studies have shown that HPV-positive cancers generally exhibit decreased expression of pRb [53]. In addition, the oncoprotein E7 of HPV is known to participate in the degradation of pRb through a ubiquitin–proteasome and other pathways [54]. Thus, the absence of HPV infection could be related to the pRb expression in our samples.

## Conclusion

We detected p16^INK4a^ in half of LSCC cases, and in half of these, HPV was also detected. However, the proportion of samples with p16^INK4a^ expression was similar between those with HPV and EBV. Although the proportion of LSCC samples with p16^INK4a^ expression was higher than reported in several previous studies, our findings suggest little value of this marker as a reliable surrogate for identifying HPV in laryngeal cancer. On the other hand, as in previous studies, most of our LSCC samples had pRb expression, this being more frequent in tumors without the presence of HPV, even in those positive for high-risk HPV. Therefore, pRb expression seems to indicate HPV negativity, which could justify not performing other molecular tests for HPV detection in this type of cases, as previously suggested. However, further studies with a larger number of cases, including controls without laryngeal cancer, involving other tumor suppression pathways or other molecular markers, are necessary to determine the real role of p16^INK4a^ and pRb in LSCC.

## Data Availability

The raw data analyzed during the current study are provided in a Supplemental File.

## References

[1] Onerci Celebi O, Sener E, Hosal S, Cengiz M, Gullu I, Guler TG (2018). Human papillomavirus infection in patients with laryngeal carcinoma. BMC Cancer.

[2] Morshed K (2010). Association between human papillomavirus infection and laryngeal squamous cell carcinoma. J Med Virol.

[3] Gama RR, Carvalho AL, Filho AL, Scorsato AP, López RVM, Rautava J (2016). Detection of human papillomavirus in laryngeal squamous cell carcinoma: systematic review and meta-analysis. Laryngoscope.

[4] Tan LSY, Fredrik P, Ker L, Yu FG, Wang DY, Goh BC (2016). High-risk HPV genotypes and P16INK4a expression in a cohort of head and neck squamous cell carcinoma patients in Singapore. Oncotarget.

[5] Chen WC, Chuang HC, Lin YT, Huang CC, Chien CY (2017). Clinical impact of human papillomavirus in laryngeal squamous cell carcinoma: a retrospective study. PeerJ.

[6] Chow LT, Broker TR (2013). Human papillomavirus infections: Warts or cancer?. Cold Spring Harb Perspect Biol.

[7] Duray A, Descamps G, Arafa M, Decaestecker C, Remmelink M, Sirtaine N (2011). High incidence of high-risk HPV in benign and malignant lesions of the larynx. Int J Oncol.

[8] Erkul E, Yilmaz I, Narli G, Babayigit MA, Gungor A, Demirel D (2017). The presence and prognostic significance of human papillomavirus in squamous cell carcinoma of the larynx. Eur Arch Oto-Rhino-Laryngol.

[9] Ayee R, Ofori MEO, Wright E, Quaye O (2020). Epstein Barr virus associated lymphomas and epithelia cancers in humans. J Cancer.

[10] Tsao SW, Tsang CM, Lo KW (2017). Epstein–Barr virus infection and nasopharyngeal carcinoma. Philos Trans R Soc B Biol Sci.

[11] Yang D, Shi Y, Tang Y, Yin H, Guo Y, Wen S (2019). Effect of HPV infection on the occurrence and development of laryngeal cancer: a review. J Cancer.

[12] Dreyer JH, Hauck F, Barros MHM, Niedobitek G (2017). PRb and cyclind1 complement p16 as immunohistochemical surrogate markers of hpv infection in head and neck cancer. Appl Immunohistochem Mol Morphol.

[13] Vats A, Trejo-Cerro O, Massimi P, Banks L (2022). Regulation of HPV E7 stability by E6-associated protein (E6AP). J Virol.

[14] Giarrè M, Caldeira S, Malanchi I, Ciccolini F, Leão MJ, Tommasino M (2001). Induction of pRb degradation by the human papillomavirus type 16 E7 protein is essential to efficiently overcome p16INK4a-imposed G1 cell cycle arrest. J Virol.

[15] Ohtani N, Brennan P, Gaubatz S (2003). Epstein–Barr virus LMP1 blocks p16INK4a-RB pathway by promoting nuclear export of E2F4/5. J Cell Biol.

[16] D’Arcangelo D, Tinaburri L, Dellambra E (2017). The role of p16INK4a pathway in human epidermal stem cell self-renewal, aging and cancer. Int J Mol Sci.

[17] Betiol JC, Sichero L, Costa HODO, De Matos LL, Andreoli MA, Ferreira S (2016). Prevalence of human papillomavirus types and variants and p16INK4a expression in head and neck squamous cells carcinomas in São Paulo, Brazil. Infect Agent Cancer.

[18] El-Naggar A, Westra W (2011). p16 expression as a surrogate marker for HPV-related oropharyngeal carcinoma: a guide for interpretative relevance and consistency. Head Neck.

[19] Jiang W, Chamberlain PD, Garden AS, Kim BYS, Ma D, Lo EJ (2018). Prognostic value of p16 expression in Epstein Bar Virus positive nasopharyngeal carcinomas. Head Neck.

[20] Vazquez-Guillen JM, Palacios-Saucedo GC, Rivera-Morales LG, Alonzo-Morado MV, Burciaga-Bernal SB, Montufar-Martinez M (2018). Infection and coinfection by human papillomavirus, Epstein–Barr virus and Merkel cell polyomavirus in patients with squamous cell carcinoma of the larynx: a retrospective study. PeerJ.

[21] Zain R, Sakamoto F, Shrestha P, Mori M (1995). Proliferating cell nuclear antigen (PCNA) expression in oral squamous cell carcinomas. Malays J Pathol.

[22] Xu L, Padalko E, Oštrbenk A, Poljak M, Arbyn M (2018). Clinical evaluation of INNO-LiPA HPV genotyping *EXTRA* II assay using the VALGENT framework. Int J Mol Sci.

[23] Kiyuna A, Ikegami T, Uehara T, Hirakawa H, Agena S, Uezato J (2019). High-risk type human papillomavirus infection and p16 expression in laryngeal cancer. Infect Agent Cancer.

[24] Mulder FJ, Klufah F, Janssen FME (2021). Presence of human papillomavirus and Epstein–Barr virus, but absence of merkel cell polyomavirus, in head and neck cancer of non-smokers and non-drinkers. Front Oncol.

[25] López López JC, Fernández Alonso N, Cuevas Álvarez J, García-Caballero T, Pastor Jimeno JC (2018). Immunohistochemical assay for neuron-specific enolase, synaptophysin, and RB-associated protein as a diagnostic aid in advanced retinoblastomas. Clin Ophthalmol.

[26] Halec G, Holzinger D, Schmitt M (2013). Biological evidence for a causal role of HPV16 in a small fraction of laryngeal squamous cell carcinoma. Br J Cancer.

[27] Herrera-gómez Á, Villavicencio-valencia V, Rascón-ortiz M, Luna-ortiz K (2009). Demografía del cáncer laríngeo en el Instituto Nacional de Cancerología. Cir Ciruj.

[28] Koirala K (2015). Epidemiological study of laryngeal carcinoma in Western Nepal. Asian Pac J Cancer Prev.

[29] Huerta AA, Rojo SA, Antonio J, Machado L (2017). Frecuencia, aspectos clínicos y factores asociados al cáncer de laringe. Acta Otorrinolaringol Cirugía Cabeza y Cuello..

[30] Orellana G MJ, Chuang Ch Á, Fulle C A, Fernández G R, Loyola B F, Imarai B C (2017). Cáncer de laringe: Serie de casos en 6 años en el Complejo Asistencial Doctor Sótero del Río. Rev Otorrinolaringol Cir Cabeza Cuello.

[31] Marioni G, Marchese-Ragona R, Cartei G, Marchese F, Staffieri A (2006). Current opinion in diagnosis and treatment of laryngeal carcinoma. Cancer Treat Rev.

[32] Gallegos-Hernandez JF, Paredes-Hernandez E, Flores-Diaz R, Minauro-Muñoz G, Apresa-Garcia T, Hernández-Hernandez DM (2007). Virus del papiloma humano asociado con cáncer de cabeza y cuello. Cir Cir.

[33] Rocha A, Bologna R, Rocha C (2012). Virus del papiloma humano y el cáncer de cabeza y cuello: revisión de la literatura desde México y Colombia. Univ Odontol.

[34] Stephen JK, Divine G, Chen KM, Chitale D, Havard S, Worsham MJ (2013). Significance of p16 in site-specific HPV positive and HPV negative head and neck squamous cell carcinoma. Cancer Clin Oncol.

[35] Young RJ, Urban D, Angel C, Corry J, Lyons B, Vallance N (2015). Frequency and prognostic significance of p16 INK4A protein overexpression and transcriptionally active human papillomavirus infection in laryngeal squamous cell carcinoma. Br J Cancer.

[36] Tong F, Geng J, Yan B, Lou H, Chen X, Duan C (2018). Prevalence and prognostic significance of HPV in laryngeal squamous cell carcinoma in Northeast China. Cell Physiol Biochem.

[37] Ben Elhadj M, Amine OEL, Mokni Baizig N, Ben Ayoub W, Goucha A, El May MV (2021). Expression profile of survivin and p16 in laryngeal squamous cell carcinoma: contribution of Tunisian patients. Ear Nose Throat J.

[38] Lazăr CS, Şovrea AS, Georgiu C, Crişan D, Mirescu ŞC, Cosgarea M (2020). Different patterns of p16INK4a immunohistochemical expression and their biological implications in laryngeal squamous cell carcinoma. Rom J Morphol Embryol.

[39] Guida M, Sanguedolce F, Bufo P (2009). Aberrant DNA hypermethylation of hMLH-1 and CDKN2A/p16 genes in benign, premalignant and malignant endometrial lesions. Eur J Gynaecol Oncol.

[40] Wong TS, Gao W, Li ZH, Chan JY, Ho WK (2012). Epigenetic dysregulation in laryngeal squamous cell carcinoma. J Oncol.

[41] Hernandez BY, Rahman M, Lynch CF, Cozen W, Unger ER, Steinau M (2016). p16(INK4A) expression in invasive laryngeal cancer. Papillomavirus Res.

[42] Sánchez Barrueco A, González Galán F, Villacampa Aubá JM, Díaz Tapia G, Fernández Hernández S, Martín-Arriscado Arroba C (2019). p16 influence on laryngeal squamous cell carcinoma relapse and survival. Otolaryngol Head Neck Surg.

[43] Dogantemur S, Ozdemir S, Uguz A, Surmelioglu O, Dagkiran M, Tarkan O (2020). Assessment of HPV 16, HPV 18, p16 expression in advanced stage laryngeal cancer patients and prognostic significance: assessment of HPV 16, HPV 18, p16 expression in laryngeal cancer. Braz J Otorhinolaryngol.

[44] Schindele A, Holm A, Nylander K, Allard A, Olofsson K (2022). Mapping human papillomavirus, Epstein–Barr virus, cytomegalovirus, adenovirus, and p16 in laryngeal cancer. Discov Oncol.

[45] Li J, Poi MJ, Tsai M-D (2011). The regulatory mechanisms of tumor supressor p16INK4 and relevance to cancer. Biochemistry.

[46] Lu DW, El-Mofty SK, Wang HL (2003). Expression of p16, Rb, and p53 proteins in squamous cell carcinomas of the anorectal region harboring human papillomavirus DNA. Mod Pathol.

[47] Morshed K, Korobowicz E, Skomra D (2008). Immunohistochemical study of retinoblastoma protein expression in laryngeal squamous cell carcinoma according to low and high overexpression. Bull Vet Inst Pulawy.

[48] Krecicki T, Smigiel R, Fraczek M, Kowalczyk M, Sasiadek MM (2004). Studies of the cell cycle regulatory proteins P16, cyclin D1 and retinoblastoma protein in laryngeal carcinoma tissue. J Laryngol Otol.

[49] Soares RC, Oliveira MC, De Souza LB, Costa ADLL, Pinto LP (2008). Detection of HPV DNA and immunohistochemical expression of cell cycle proteins in oral carcinoma in a population of Brazilian patients. J Appl Oral Sci.

[50] Langendijk JA, Psyrri A (2010). The prognostic significance of p16 overexpression in oropharyngeal squamous cell carcinoma: implications for treatment strategies and future clinical studies. Ann Oncol.

[51] Shaikh MH, Khan AI, Sadat A, Chowdhury AH, Jinnah SA, Gopalan V (2017). Prevalence and types of high-risk human papillomaviruses in head and neck cancers from Bangladesh. BMC Cancer.

[52] Nemes JA, Deli L, Nemes Z, Márton IJ (2006). Expression of p16INK4A, p53, and Rb proteins are independent from the presence of human papillomavirus genes in oral squamous cell carcinoma. Oral Surg Oral Med Oral Pathol Oral Radiol Endod.

[53] Wiest T, Schwarz E, Enders C, Flechtenmacher C, Bosch FX (2002). Involvement of intact HPV16 E6/E7 gene expression in head and neck cancers with unaltered p53 status and perturbed pRb cell cycle control. Oncogene.

[54] Fiedler M, Müller-Holzner E, Viertler HP (2004). High level HPV-16 E7 oncoprotein expression correlates with reduced pRb-levels in cervical biopsies. FASEB J.

